# Protein intakes of pregnant women and children in India—protein quality implications

**DOI:** 10.1111/mcn.12952

**Published:** 2020-12-21

**Authors:** Sulagna Bandyopadhyay, Nirupama Shivakumar, Anura V. Kurpad

**Affiliations:** ^1^ Division of Nutrition, St. John's Research Institute St. John's National Academy of Health Sciences Sarjapur Road, Bangalore 560034 India; ^2^ Department of Physiology, St. John's Medical College St. John's National Academy of Health Sciences Sarjapur Road, Bangalore 560034 India

**Keywords:** child growth, infant and child nutrition, low birthweight, nutrition–infection interaction, pregnancy and nutrition, stunting, undernutrition

## Abstract

The recent National Family Health Survey (NFHS‐4, 2016) reports a national average of 18% for low birthweight (LBW) and 38% for stunting in children <5 years. Nutrition and environmental influences (chronic enteric pathogenic exposure through poor water, sanitation, and hygiene) are two critical factors that impact the health outcomes of the populxation. This is particularly relevant for vulnerable age groups such as pregnant women and children <5 years, who bear long‐lasting and intergenerational consequences of impoverished nutrition and suboptimal living conditions. The present review provides, for the first time, an analysis of indispensable amino acid (IAA) requirements for pregnant women, separately for the second and third trimesters, using protein accretion data from a recent Indian study. Furthermore, using these estimates for pregnancy, and the current IAA requirements for young children, the quality of protein was assessed in Indian diets consumed by pregnant women and children (1–3 and 4–6 years) from national representative rural National Nutrition Monitoring Bureau survey. The assessment was considered in the context of an adverse environment and in relation to outcomes such as LBW, stunting, and underweight. Finally, an assessment was made of the proportion of the surveyed population at risk of dietary quality protein inadequacy and implications for planning nutrition intervention programmes. Specifically, state‐wise estimates of the risk of quality protein inadequacy are provided, in addition to evaluations of additional dietary supplementation, which could inform the policy of supplementary nutrition programmes to improve health outcomes.

**Key messages**

- Trimester‐specific (second and third) indispensable amino acid requirements are provided for pregnant women and should be used for protein quality assessment of their dietary intakes.
- The protein quality of dietary intakes in children (1–3 and 4–6 years) from 10 Indian states of the rural National Nutrition Monitoring Bureau survey showed a significant inverse association with the state‐wise prevalence of stunting and underweight.
- Supplementary food intake through nutrition programmes should be optimised for its protein quality, by using either animal source foods or a legume/cereal mixture along with animal source foods.

## 1 INTRODUCTION

The prevalence of poor birth outcomes and chronic undernutrition continues to be high in low‐ and middle‐income countries, with India reporting a national average of 18% low birthweight (LBW) and 38% stunting in children <5 years (National Family Health Survey‐4, 2016). Diets in India are predominantly cereal based and lack diversity (Agrawal et al., 2019; Nithya & Bhavani, 2018), which increases the risk of macronutrient and micronutrient inadequacy in its population. This risk of inadequate dietary intake could specifically impact vulnerable populations such as pregnant women and children, leading to poor outcomes of birth and nutritional status (Ghosh, 2016). Protein‐rich foods are also a good source of diverse nutrients, and surveys consistently show that overall nutrient adequacy of a diet improves with inclusion of such foods from various sources (de Gavelle, Huneau, & Mariotti, 2018; Phillips et al., 2015).

Dietary proteins, particularly its constituent indispensable amino acids (IAAs), are required in adequate quantity and proportion to allow for body tissue protein synthesis along with various enzymatic and structural activities (Wu, 2010). Specifically, IAAs are essential for maternal health, in promoting positive birth outcomes (Ghosh, 2016), supporting linear growth in young children (Millward, 2017), and maintaining tissue health, specifically skeletal muscle, in adults and the elderly (Burd, McKenna, Salvador, Paulussen, & Moore, 2019; Franzke, Neubauer, Cameron‐Smith, & Wagner, 2018). The joint World Health Organization, Food and Agriculture Organization & United Nations University (2007) consultation has revised the daily protein requirement for pregnant women and children from its earlier recommendations (Food and Agricultural Organization, World Health Organization & United Nations University, 1985).

Although recent national dietary surveys in India show that the daily total protein intake is adequate (National Sample Survey Office, 2014), these estimates are based on the protein density in foods, without considering the digestibility and availability of IAA from these foods. To assess the IAA availability from the diet, a protein quality score needs to be calculated, which depends on the IAA composition of a food in relation to the age‐specific daily IAA requirement (the amino acid score), as well as the specific IAA digestibility of the food, as measured in humans (Food and Agricultural Organization (FAO), 2013). The available protein quality scoring methods are the protein digestibility‐corrected amino acid score (PDCAAS), which uses a common oro‐faecal balance‐based digestibility factor for all IAA, and the digestible indispensable amino acid score (DIAAS), which is a more accurate metric of protein quality, as it uses digestibility factors that are based on digestion and absorption at an ileal level and is specific for each IAA (FAO, 2013). In addition, the effect of the environment on the digestive and absorptive functions of the intestine should be considered (Kurpad, 2006) when defining the IAA requirement, which will in turn impact its scoring pattern. This is of particular relevance to young children (<5 years), wherein, when safe environmental conditions were ensured, high quality protein intake was shown to be more efficacious for linear growth (Millward, 2017). This review will discuss the IAA requirements for pregnant women and children <5 years, protein quality (DIAAS) assessment of their diets in relation to nutritional outcomes from a national representative rural survey (National Nutrition Monitoring Bureau, 2012), environmental influence on the IAA requirements, and the implications of protein quality and environment on planning the provision of protein sources for nutrition intervention programmes.

## 2 METHODS

### 2.1 IAA requirements in children and pregnant women

The current daily estimated average requirement (EAR) of IAA for children was derived from a factorial approach, which is a sum of the mean maintenance IAA requirement and IAA requirement for growth. For the growth component, the protein deposited during growth corrected for efficiency of utilisation is used (World Health Organization, Food and Agricultural Organization & United Nations University, 2007). The scoring pattern of the requirement is then calculated as a ratio of total IAA requirement (mg·kg^−1^·day^−1^) to the calculated growth plus maintenance protein requirement (g·kg^−1^·day^−1^; World Health Organization, Food and Agricultural Organization & United Nations University, 2007). The factorial estimates of IAA requirement (mg·kg^−1^·day^−1^) compared well with empirical estimates of the requirement for some IAA in Indian children, using the stable isotope‐based indicator amino acid oxidation method (Kurpad & Thomas, 2011; Pillai & Kurpad, 2012).

The present additional daily protein requirements during the trimesters of pregnancy are based on measurements of total body potassium accretion in well‐nourished mothers from a high‐income country (World Health Organization, Food and Agricultural Organization & United Nations University, 2007). It is important to note that the World Health Organization, Food and Agricultural Organization & United Nations University, 2007 additional protein requirement of pregnant women implies the intake of high‐quality protein, which could supply all the required additional IAA for optimal foetal growth. However, the report did not explicitly comment on the total or additional daily IAA requirement of pregnant women. It is therefore difficult to specifically evaluate the diets of pregnant women for their protein quality; this is relevant for operationalising feeding and subsidy programmes.

To compute the additional daily IAA requirement during pregnancy (separately for second and third trimesters), a factorial approach was used, with recent estimations of total body potassium accretion based additional protein requirements in well‐nourished, middle socio‐economic stratum South Indian pregnant women with a total gestational weight gain (GWG) of 10.7 kg (Kuriyan et al., 2019). In the present analyses, using the factorial approach, the additional IAA requirements were calculated as a sum of IAA requirements for growth (newly deposited tissue) and maintenance of the total GWG (including maintenance of the newly deposited tissue). Therefore, first, the additional protein requirement (g·kg^−1^·day^−1^) for growing tissue (foetal and maternal tissue deposition) that had been adjusted for an efficiency of utilisation of 42% (World Health Organization, Food and Agricultural Organization & United Nations University, 2007) was multiplied by the IAA composition (mg g^−1^ protein) of foetal tissue (Widdowson, 1980). Because foetal protein accounts for a major portion of protein accretion (42%; Rasmussen & Yaktine, 2009), and IAA composition of different maternal tissues were not available, it was reasonable to use IAA composition of foetal tissue (Widdowson, 1980) to represent the overall tissue deposition. Moreover, IAA composition of mixed (World Health Organization, Food and Agricultural Organization & United Nations University, 2007) and foetal tissue (Widdowson, 1980) are reasonably similar in humans. Second, the GWG maintenance protein requirement (g·kg^−1^·day^−1^) was multiplied by the maintenance IAA requirement pattern (mg g^−1^ protein) for adults (World Health Organization, Food and Agricultural Organization & United Nations University, 2007). In the foetal tissue IAA composition (Widdowson, 1980), the concentration of tryptophan and cysteine was not available; for the sulphur containing amino acids (SAA), a 1:1 ratio of methionine: cysteine was assumed. For the second and third trimesters, the additional IAA requirements for tissue deposition were calculated separately using mean values of foetal tissue IAA composition from 12–24 and 28–40 weeks, respectively (Widdowson, 1980). The scoring pattern was calculated as the ratio of the body weight‐corrected IAA requirement (mg·kg^−1^·day^−1^) and the body weight‐corrected total protein requirement (g·kg^−1^·day^−1^) for the pregnant women studied (Kuriyan et al., 2019).

### 2.2 Protein quality assessment (DIAAS) of supplementary food sources to meet the additional protein and IAA requirement in the second and third trimesters of pregnancy

The IAA scores of foods contributing to protein in the daily Indian diet were calculated as the ratio of the IAA scores of food protein (mg g^−1^ protein) and the IAA requirement scoring pattern (mg g^−1^ protein) of the second and third trimester additional protein requirement. Food composition values were taken from the *Indian food composition tables* (Longvah, Anantan, Bhaskarachary, & Venkaiah, 2017). These scores were adjusted for the true ileal IAA digestibility values of a representative food (Table 1), to estimate the digestible indispensable amino acid reference ratio (DIAA reference ratio) and the DIAAS of these foods. The details of DIAAS calculation for individual foods (as supplementary food sources) are previously reported (FAO, 2013). Recently, a series of studies were conducted in South Indian adults and children (<2 years) to generate true ileal IAA digestibility values for commonly consumed animal (hen's egg and meat) and plant (rice, ragi, *desi* chickpea, *kabuli* chickpea, yellow pea, and mung bean) protein source foods, using the dual stable isotope technique. In the present analysis, the true ileal IAA digestibility values (from adult studies) of whole mung bean, hen's egg, and meat (Kashyap et al., 2018; Kashyap et al., 2019) were used to calculate the DIAAS of supplementary food sources, that is, legume, egg, and meat, respectively, for pregnant women. For cereal protein, the true ileal IAA digestibility of rice was used from the study on children <2 years (Shivakumar et al., 2019), whereas the digestibility of cow's milk was assumed to be the same as hen's egg protein from the study on adults (Kashyap et al., 2018).

**TABLE 1:** Mean true ileal IAA digestibility (%) of commonly consumed plant and animal proteins in South Indians

IAA	Adults^a^						Children^b^			
	Whole mung bean	De‐hulled mung	Yellow pea	Chick pea (*kabuli*)	Whole egg	Chicken meat	Rice	Finger millet	Mung bean	Egg
Methionine	52.2	64.3	56.1	71.8	85.9	92.7	79.7	60.1	54.0	85.9
Phenylalanine	73.4	75.1	80.6	80.9	95.6	94.4	83.9	69.4	77.2	89.6
Threonine	42.5	54.5	66.8	72.5	96.2	93.7	73.4	67.2	61.6	90.1
Lysine	63.0	63.4	62.1	60.0	88.9	95.5	78.3	74.5	64.8	93.6
Leucine	67.5	76.3	79.0	79.5	87.6	89.1	78.7	67.2	68.0	86.3
Isoleucine	75.8	82.9	79.5	81.4	85.4	88.8	80.5	75.4	63.0	85.4
Valine	67.8	80.0	77.4	75.9	86.6	89.6	75.2	65.3	68.0	81.2
Mean IAA	63.2	70.9	71.6	74.6	89.4	92.0	78.5	68.4	65.2	87.4

Abbreviation: IAA, indispensable amino acid.

^a^ Kashyap et al., 2018; Kashyap et al., 2019.

^b^ Shivakumar et al., 2019.

### 2.3 Adverse environmental influence on IAA requirements of children and pregnant women

Developing countries share greater than 95% of the global burden of stunting in children <5 years (De Onis, Blössner, & Borghi, 2012). This is attributed to the suboptimal quality protein intake and poor environmental conditions, which includes chronic pathogenic exposure (bacterial and parasitic) compounded by family and societal stresses (Crane, Jones, & Berkley, 2015). Prolonged exposures to an impoverished environment can alter the enzymatic secretions in gut, leading to compromised digestion and absorption of the nutrients (Banwell et al., 1967). If the digestion and absorption of protein is low, it is likely that the protein and IAA requirements (especially the limiting IAA, such as lysine) of individuals from poor socio‐economic environments will be higher than the recommendations made for a healthy population from safe or “clean” environments. This has been supported by a study on chronically undernourished men from poor socio‐economic conditions, where daily requirement of lysine was found to be higher by ~50% compared with their age‐matched controls (Kurpad et al., 2003). Such increase in the requirement could partly be attributed to the parasitic infections, as a subsequent deworming trial on school children from compromised environmental conditions showed that their lysine requirement was ~20% higher in the presence of parasites (Pillai, Elango, Ball, Kurpad, & Pencharz, 2015). Although it is difficult to provide an accurate estimate of the additional IAA requirement imposed by the environmental stress, a potential increase in the EAR of IAA is a necessary consideration. This is of relevance in the current context as the latest NNMB survey (National Nutrition Monitoring Bureau, 2012) reported that 66% of the households in rural India are not using or do not have access to sanitary latrine facility and 20% are deprived of safe drinking water as per the recommended choices of improved drinking water sources (World Health Organization, 2017), suggesting that the population is exposed to unhygienic environmental conditions. Therefore, a 20% increase was considered for the daily lysine (limiting IAA in the Indian diet) requirement of children 1–3 and 4–6 years to calculate DIAAS of their dietary intakes from the rural NNMB survey (National Nutrition Monitoring Bureau, 2012).

Chronic enteric pathogenic exposure leading to subclinical environmental enteropathy in pregnant women has been observed to affect birth outcomes (Lauer et al., 2018). The researchers reported elevation in anti‐flagellin and anti‐lipopolysaccharide immunoglobulin concentrations, which reflect possible enteropathy, to be associated with shorter gestation and lower birth lengths. This was the first study to show the probability of an environmental impact on pregnancy that in turn relates to adverse birth outcomes (Lauer et al., 2018). A study on Indian, Caribbean, and U.S. women showed that the lactulose: mannitol ratio tended to be higher in Indian non‐pregnant non‐lactating women, with a lower net mannitol absorption (Kao et al., 2015), suggesting that they could have some intestinal dysfunction and consequently a lower protein digestion and absorption. However, for pregnancy, an increased IAA requirement was not used to calculate DIAAS, because of the lack of data on the environmental interactions and some evidence on the increased efficiency of lysine (and perhaps other IAA) utilisation (by ~10%) in pregnant pigs for the second and third periods of gestation (Navales et al., 2019), although there are no similar studies in humans.

### 2.4 Protein quality evaluation in relation to nutritional outcomes and the risk of protein inadequacy in children and pregnant women from rural India

The relation between dietary protein quality (DIAAS) and nutritional outcomes was evaluated in a representative rural population of Indian children (1–3 and 4–6 years) and pregnant women, using dietary intake data from the latest NNMB survey in 10 Indian states; the detailed methodology of the survey is provided elsewhere (National Nutrition Monitoring Bureau, 2012). For the purpose of the DIAAS calculation in children 1–3 years, the daily IAA requirements were calculated using a mean growth protein requirement (for ages 1, 1.5, 2, 2.5, and 3 years) multiplied by the mixed tissue IAA pattern and added to the maintenance IAA requirement (as per the factorial method from World Health Organization, Food and Agricultural Organization & United Nations University, 2007). The scoring pattern was calculated as the ratio of the IAA requirement (mg·kg^−1^·day^−1^) and total (mean growth plus maintenance) protein requirement (g·kg^−1^·day^−1^). For children 4–6 years, the IAA requirements and scoring pattern for the age group of 3–10 years was used (adapted from World Health Organization, Food and Agricultural Organization & United Nations University, 2007). For pregnant women, one problem was that the specific trimester during which dietary data were collected was not documented in the NNMB report (NNMB, 2012); therefore, an average protein and IAA requirement (for both the trimesters) was considered for the purpose of dietary evaluation. Thus, a mean pregnancy protein requirement (referred as mid‐pregnancy protein requirement) of 0.87 g·kg^−1^·day^−1^ was calculated by adding the maintenance protein requirement of 0.66 g·kg^−1^·day^−1^ to a mean value of 0.21 g·kg^−1^·day^−1^ for GWG (based on a mid‐pregnancy GWG of 5.3 kg and first trimester weight of 57.8 kg). Similarly, a mean pregnancy daily IAA requirement was calculated by adding the mean additional IAA requirement (referred as mid‐pregnancy IAA requirement) for maintenance and foetal growth (average of the second and third trimesters) to the daily maintenance IAA requirement of the non‐pregnant non‐lactating woman. Finally, the scoring pattern (mg g^−1^ protein) was calculated as a ratio of mid‐pregnancy IAA and protein requirement.

The DIAAS was calculated for children aged 1–3 and 4–6 years and pregnant women based on the food intake (g day^−1^) records of 10 states, using IAA digestibility values (Table 1) of one representative food from each food group. In the present analysis, four out of seven food groups, namely, cereals and millets, legumes, milk and milk products, and other animal source food (ASF; fish, egg, meat, and poultry), that contribute predominantly to the daily protein intake were selected. The representative foods were rice for cereals, finger millet for millets, whole mung bean for legumes, and hen's egg for ASFs (Kashyap et al., 2018; Kashyap et al., 2019; Shivakumar et al., 2019). The details of DIAAS calculation has been reported earlier (FAO, 2013; Shivakumar et al., 2019).

Spearman's correlations were performed to evaluate the association of the state‐wise DIAAS of the diet with different nutritional outcomes, such as the prevalence of stunting and underweight in children 1–3 and 4–6 years and LBW prevalence for pregnant women. The risk of quality protein inadequacy in children (1–3 and 4–6 years) and pregnant women was estimated by the EAR cut‐point method (Institute of Medicine Subcommittee on Interpretation and Uses of Dietary Reference Intakes; Institute of Medicine Standing Committee on the Scientific Evaluation of Dietary Reference Intakes, 2000), for crude and DIAAS‐corrected protein intake and their reported distribution, using the EAR for protein as described above. For children aged 1–3 and 4–6 years, the EAR of protein (0.81 and 0.73 g·kg^−1^·day^−1^; World Health Organization, Food and Agricultural Organization & United Nations University, 2007) was adjusted upward by 20% (0.97 and 0.88 g·kg^−1^·day^−1^) to account for environmental stress.

## 3 RESULTS

The additional IAA requirements (mg·kg^−1^·day^−1^) and scoring patterns (mg g^−1^) are presented in Table 2. The DIAA reference ratio and DIAAS values of individual foods (as supplementary food sources) for pregnant women, separately for the second and third trimesters, are provided in Table 3. ASFs showed a high DIAAS of ≥90% in both the trimesters, whereas it was <45% for the plant‐based foods. Lysine and SAA were the limiting IAAs in cereals and legumes (with lowest DIAA reference ratio), respectively, and defined DIAAS of these supplementary food sources. This suggests that only ASFs like egg, milk and milk products, and meat can meet the additional quality protein requirement of pregnancy; whereas others, like plant/legume sources, would have to be consumed in greater quantities. For example, to meet the second trimester quality‐corrected protein requirement (~8 g day^−1^), the pregnant women's diet should include an extra 250 ml of milk (2½ glasses) or 70 g of egg (1½ eggs) or 120 g of legumes. As an additional intake, the quantity of legumes appears to be daunting but can be more reasonable when consumed in combination with ASF, for example, 30 g of legumes along with either 100 ml of milk or 25 g of egg, or a mix of 50‐g legume and 50‐g rice.

**TABLE 2:** Additional protein requirement, IAA requirement, and IAA scoring pattern for pregnant women

Trimester	Additional protein requirement (g·kg^−1^·day^−1^)		Additional IAA requirement^a^ (mg·kg^−1^·day^−1^)						
	Maintenance^b^	Foetal growth^c^	Ile	Leu	Lys	SAA	AAA	Thr	Val
Second	0.03	0.11	5	10	9	5	9	5	6
Third	0.08	0.21	10	21	19	10	18	11	13
			IAA requirement scoring pattern^d^ (mg g^−1^ protein)						
Second			34	71	66	34	65	37	44
Third			35	72	66	35	63	37	46

Abbreviations: AAA, aromatic amino acids; IAA, indispensable amino acid; Ile, isoleucine; Leu, leucine; Lys, lysine; SAA, sulphur amino acids; Thr, threonine; Val, valine.

^a^ Based on the sum of the product of the maintenance protein requirement and the maintenance IAA pattern and the product of protein deposition for foetal growth and the IAA composition of the fetus.

^b^ Maintenance protein requirement of the gestational weight gain over the second and third trimesters, expressed per kilogram body weight per day. Body weight was calculated as sum of end of first trimester body weight and second or third trimester gestational weight gain (Kuriyan et al., 2019).

^c^ Additional protein requirement for foetal growth at each trimester, expressed per kilogram body weight per day (body weight was calculated as described above; Kuriyan et al., 2019).

^d^ Scoring pattern calculated as the ratio of additional requirement of each IAA (mg·kg^−1^·day^−1^) and the additional protein requirement (g·kg^−1^·day^−1^).

**TABLE 3:** Protein quality (DIAAS) of supplementary food sources for pregnant women at the second and third trimesters

Foods	Digestible indispensable amino acid reference ratio for individual food protein sources^a^ (%)							DIAAS (%)^b^
	Ile	Leu	Lys	SAA	AAA	Thr	Val	
	Second trimester							
Cereal	102	91	44	104	126	64	104	44
Legume	94	72	63	30	102	37	85	30
Milk	155	133	117	95	156	123	127	95
Egg	137	91	111	426	152	164	119	91
Meat	117	102	108	422	153	139	97	97
	Third trimester							
Cereal	101	88	44	101	129	65	100	44
Legume	92	70	63	29	105	37	81	29
Milk	153	130	116	92	161	124	121	92
Egg	135	89	110	414	157	166	114	89
Meat	116	100	107	411	157	141	93	93

Abbreviations: AAA, aromatic amino acids; DIAAS: digestible indispensable amino acid score; Ile, isoleucine; Leu, leucine; Lys, lysine; SAA, sulphur amino acids; Thr, threonine; Val, valine.

^a^ Digestible indispensable amino acid reference ratio was calculated based on true ileal digestibility values of foods in Indians (Kashyap et al., 2018; Kashyap et al., 2019; Shivakumar et al., 2019). For this calculation, each food group was represented by a single food. Cereal: rice; legume: whole mung bean; milk: cow milk (whole); egg: hen egg; meat: chicken skeletal muscle. Amino acid composition of foods was taken from the *Indian food composition tables* (Longvah et al., 2017).

^b^ DIAAS was represented by the lowest value of the digestible indispensable amino acid reference ratio (%) in each supplementary food source.

The mean daily estimated average protein requirement (g·kg^−1^·day^−1^), IAA requirement (mg·kg^−1^·day^−1^), and scoring pattern (mg g^−1^ protein) for children 1–3 and 4–6 years and pregnant women are presented in Table 4. The DIAAS, crude, and DIAAS‐corrected protein intake data of 10 states are presented for children 1–3 and 4–6 years in Table 5 and for pregnant women in Table 6. The DIAAS estimates for pooled dietary intake in children 1–3 and 4–6 years and pregnant women were 69%, 66%, and 74%, respectively. Lowest and highest estimates of DIAAS were observed in Madhya Pradesh and Kerala, respectively, for both children (1–3 and 4–6 years) and pregnant women. Lysine was the limiting IAA in the diets of both population groups across all the states.

**TABLE 4:** Protein requirement, IAA requirement, and scoring pattern for children 1–3 and 4–6 years and pregnant women (during the second and third trimesters)

	Protein requirement (g·kg^−1^·day^−1^)		IAA requirement (mg·kg^−1^·day^−1^)			
	Maintenance^b^	Growth^c^	Lys	SAA	AAA	Thr
1–3 years^a^	0.66	0.15	49	20	36	22
4–6 years^b^	0.66	0.07	42	17	30	18
Mid‐pregnancy^c^	0.66	0.21	44	22	39	23
			IAA scoring pattern^d^ (mg g^−1^ protein)			
1–3 years			60	24	45	27
4–6 years			57	23	41	25
Mid‐pregnancy			50	25	44	26

Abbreviations: AAA, aromatic amino acids; IAA, indispensable amino acid; Lys, lysine; SAA, sulphur amino acids; Thr, threonine.

^a^ Mean growth protein requirement (g·kg^−1^·day^−1^) was calculated as an average growth requirement of children 1, 1.5, 2, 2.5, and 3 years from WHO (2007); maintenance protein requirement (g·kg^−1^·day^−1^) was taken from WHO (2007); daily lysine requirement (mg·kg^−1^·day^−1^) was increased by 20% to account for environmental stress.

^b^ Protein requirement (g·kg^−1^·day^−1^) for growth and maintenance was reproduced from the requirements of 3–10 years (WHO, 2007); daily lysine requirement (mg·kg^−1^·day^−1^) was increased by 20% to account for environmental stress.

^c^ Daily IAA requirements were calculated based on the sum of the (a) product of the mean second and third trimester pregnancy maintenance protein requirement and the maintenance IAA pattern and (b) the product of the mean second and third trimester protein deposition for foetal growth and the IAA composition of the fetus; additional mean second and third trimester protein requirement (mg·kg^−1^·day^−1^) for foetal growth was calculated using mid‐pregnancy body weight, taken as a sum of body weight at the end of the first trimester and half the gestational weight gain over the second and third trimesters (Kuriyan et al., 2019); maintenance protein requirement of non‐pregnant non‐lactating women was taken from WHO (2007).

^d^ Scoring pattern of IAA requirement (mg g^−1^ protein) for children 1–3 and 4–6 years and pregnant women was calculated as a ratio of each IAA requirement (mg·kg^−1^·day^−1^) and the protein requirement (g·kg^−1^·day^−1^).

**TABLE 5:** Dietary crude protein intake, DIAAS of the diets, DIAAS‐corrected protein intake, and risk of crude and DIAAS‐corrected protein inadequacy in children aged 1–3 and 4–6 years across states from NNMB rural survey data

States	1–3 years					4–6 years				
	Crude protein intake^a^ (g day^−1^)	DIAAS^b^ (%)	DIAAS‐corrected protein intake^c^ (g day^−1^)	Risk of dietary crude protein inadequacy^d^ (%)	Risk of DIAAS‐corrected protein inadequacy^d^ ^,^ ^e^ (%)	Crude protein intake^a^ (g day^−1^)	DIAAS^b^ (%)	DIAAS‐corrected protein intake^c^ (g day^−1^)	Risk of dietary crude protein inadequacy^d^ (%)	Risk of DIAAS‐corrected protein inadequacy^d^ ^,^ ^e^ (%)
Kerala	17.2	85	14.7	30.2	43.5	26.6	83	22.2	18.6	33.5
Tamil Nadu	21.0	86	18.1	15.8	30.0	27.8	81	22.5	15.4	32.1
Karnataka	23.5	64	14.9	14.1	42.2	29.1	63	18.4	10.3	41.9
Andhra Pradesh	18.7	69	13.0	21.7	48.5	25.6	69	17.7	13.7	43.4
Maharashtra	18.4	72	13.2	24.2	47.7	26.1	69	17.9	15.9	43.4
Gujarat	21.5	62	13.4	19.6	47.5	34.5	64	22.1	10.3	35.4
Madhya Pradesh	23.3	58	13.6	13.9	46.5	35.6	59	20.9	3.2	33.5
Orissa	19.3	60	11.5	16.6	54.5	26.2	62	16.1	10.0	48.6
West Bengal	20.8	80	16.6	17.0	35.6	23.8	70	16.8	14.3	46.1
Uttar Pradesh	26.5	71	18.7	6.9	28.5	38.6	63	24.3	7.2	31.3
Pooled	21.3	69	14.8	18.1	42.6	30.3	66	20.1	12.0	38.3

Abbreviations: DIAAS, digestible indispensable amino acid score; NNMB, National Nutrition Monitoring Bureau.

^a^ Mean crude protein (g day^−1^) intake values are taken from NNMB survey data (NNMB, 2012).

^b^ DIAAS was calculated with 20% increase in lysine requirement to compensate for the extra demand due to poor environment.

^c^ DIAAS‐corrected protein intake (g day^−1^) = Crude protein intake (g day^−1^) × DIAAS (%)/100.

^d^ Risk of dietary crude protein inadequacy and risk of DIAAS‐corrected protein inadequacy were calculated based on estimated average requirement cut‐point method (Institute of Medicine, 2000) in NNMB rural survey data (NNMB, 2012).

^e^ Risk was calculated with a 20% increase in estimated average requirement of protein to account for the increased demand of protein due to poor environmental conditions.

**TABLE 6:** Dietary crude protein intake, DIAAS of the diets, DIAAS‐corrected protein intake, and risk of crude and DIAAS‐corrected protein inadequacy in pregnant women across states from NNMB rural survey data

States	Pregnant women				
	Crude protein intake^a^ (g day^−1^)	DIAAS (%)	DIAAS‐corrected protein intake^b^ (g day^−1^)	Risk of inadequacy from crude protein intake^c^ (%)	Risk of inadequacy form DIAAS‐corrected protein intake^c^ (%)
Kerala	54.7	94	51.3	45.5	50.4
Tamil Nadu	42.6	79	33.7	77.1	93.0
Karnataka	47.2	70	32.9	58.5	81.9
Andhra Pradesh	40.8	73	29.9	73.9	90.0
Maharashtra	34.8	68	23.7	93.7	99.4
Gujarat	61.4	75	46.1	32.7	60.0
Madhya Pradesh	61.4	65	40.0	25.0	78.7
Orissa	48.3	67	32.1	62.9	97.4
West Bengal	35.7	71	25.2	97.9	100.0
Uttar Pradesh	59.4	70	41.4	39.6	63.4
Pooled	48.6	74	35.8	55.5	76.9

Abbreviations: DIAAS, digestible indispensable amino acid score; NNMB, National Nutrition Monitoring Bureau.

^a^ Mean crude protein (g day^−1^) intake values are taken from NNMB data (NNMB, 2012).

^b^ DIAAS‐corrected protein intake (g day^−1^) = Crude protein intake (g day^−1^) × DIAAS (%)/100.

^c^ Risk of dietary crude protein inadequacy and risk of DIAAS‐corrected protein inadequacy were calculated based on estimated average requirement cut‐point method (Institute of Medicine, 2000) in NNMB rural survey data (NNMB, 2012); estimated average requirement of protein (g·kg^−1^·day^−1^) for pregnant women was taken as 0.87 g·kg^−1^·day^−1^ (addition of protein requirement for maintenance and foetal growth) from Table 3, which was multiplied by body weight of pregnant women. For the body weight, half the gestational weight gain (GWG) of 4 kg (below) was added to the non‐pregnant non‐lactating reference body weight of 55 kg to calculate total protein requirement and risk of protein inadequacy in diets; a GWG of 8 kg was previously reported (National Nutrition Monitoring Bureau, 1984); however, in the absence of data on the trimester of recruitment in the latest NNMB survey (NNMB, 2012), the reported GWG was halved (4 kg).

**FIGURE 1:**
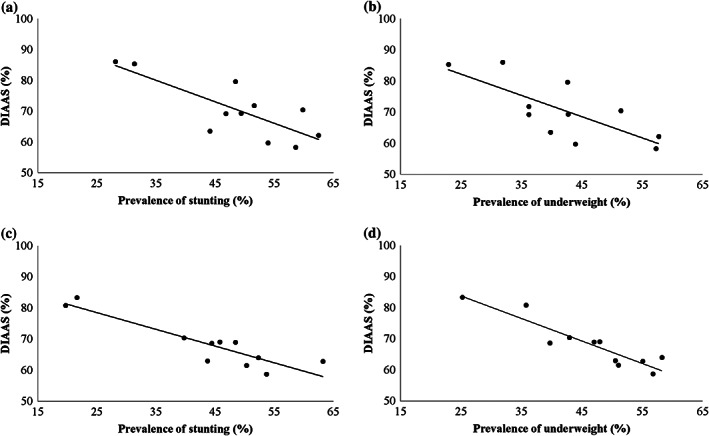
Relation between digestible indispensable amino acid score (DIAAS) and prevalence of stunting and underweight in children (a and b) 1–3 years and (c and d) 4–6 years from 10 states of rural India; *r*s *=* −.63 (*P* < .05), *r*s *=* −.75 (*P* < .01), *r*s = −.80 (*P* < .01), and *r*s = −.88 (*P* < .01) for (a), (b), (c), and (d), respectively. Data were taken from the latest rural National Nutrition Monitoring Bureau survey (NNMB, 2012)

State‐level analyses showed an inverse and significant correlation (Figure 1) between the protein quality estimate (DIAAS) of the children's dietary intakes and prevalence of stunting and underweight in 1–3 (*r*s = −.63, *P <* .05 and *r*s = −.75, *P* < .01, respectively) and 4–6 years (*r*s = −.80, *P <* .01 and *r*s = −.88, *P* < .01, respectively). A similar inverse but non‐significant (possibly due to the small sample size) association was observed between the DIAAS of the pregnancy diets and the prevalence of LBW (*r*s = −.53, *P* = .09). Finally, the risk of crude and DIAAS‐corrected protein inadequacy in children 1–3 and 4–6 years as well as pregnant women across different Indian states is presented in Tables 5 and 6, respectively. The estimate for risk of crude protein inadequacy, for pooled dietary intake, in children 1–3 and 4–6 years and pregnant women were 18%, 12%, 56%, which increased to 43%, 38%, and 77% after DIAAS correction. The highest risk of DIAAS‐corrected protein intake was observed in Orissa and West Bengal for children (1–3 and 4–6 years) and pregnant women, respectively, whereas the lowest was observed in Uttar Pradesh and Kerala.

## 4 DISCUSSION

The importance of assessing protein quality of foods used in supplementation and regular consumption, particularly in vulnerable populations, has been constantly impressed upon by the expert consultations (World Health Organization, Food and Agricultural Organization & United Nations University, 2007; FAO, 2013; FAO, 2014). The need for replacing the PDCAAS with DIAAS on availability of appropriate ileal digestibility values was also proposed (FAO, 2014). DIAAS assessment is now possible in humans, because of the recently generated true ileal IAA digestibility values for commonly consumed foods in Indian meal matrices (Kashyap et al., 2018; Kashyap et al., 2019; Shivakumar et al., 2019). Furthermore, the issue of chronic environmental pathogenic exposure leading to impaired intestinal function needs consideration. A study on a group of healthy children from West Africa (Dakar and Abidjan province) showed a significantly lower concentration of all pancreatic enzymes, electrolytes, and water compared with their age‐ and sex‐matched controls from France (Marseille province), in duodenal aspirates after pancreatic stimulation by pancreozymin (Sauniere & Sarles, 1988). The study also reported an elevated lactoferrin levels in the children from West Africa, which indicated a subclinical pancreatic damage and demonstrated a silent pancreatic insufficiency in the population (Sauniere & Sarles, 1988). In addition, adverse environmental exposure leads to morphological changes in the intestinal epithelium such as villous atrophy, low villus to crypt ratio, increased epithelial permeability, and inflammation (Owino et al., 2016). This impairs absorption and causes subsequent nutrient loss from the gastrointestinal tract (Keusch et al., 2014). Further, chronic stimulation of immune system by enteric pathogens may lead to nutrient partitioning towards supporting the immune response, leaving less available for growth and maintenance. A murine model showed that a poor diet with low protein and fat content (7% and 5%, respectively), compounded by an environmental pathogenic insult (from a bacterial pathogen cocktail), developed features of enteric dysfunction within 3 weeks with altered small intestinal microbiota, permitting greater colonisation of enteric pathogens and increasing susceptibility to enteric infections (Brown et al., 2015).

The recent plant protein IAA digestibility values from Indian studies (Kashyap et al., 2018; Kashyap et al., 2019; Shivakumar et al., 2019) when utilised for DIAAS assessment suggest that cereal : legume complementation may not be sufficient to optimally improve protein quality in children and pregnant women; in this specific instance, an increase in food protein quantity is required but with the risk of excess energy intake. As the volume of food also increases, this will be difficult to achieve in young children with relatively low gastric capacity. The national Indian flagship supplementary nutrition programmes (such as Integrated Child Development Scheme and Mid‐day Meal Scheme) to vulnerable populations presently provide 600 kcal day^−1^ to pregnant and lactating women. This contains 20 g of protein but comes from a cereal: legume mixture, without consideration of the biological availability of protein or additional allowances that could be made for a poor environment. Similar considerations are taken for the provision of subsidy to young children, either through take‐home rations or meals provided in community creches (Anganwadi). However, sporadic but laudatory efforts have begun in some states to provide egg and milk through hot cooked meals at Anganwadi. This is relevant, as intervention trials with plant‐based protein supplementation show suboptimal effectiveness on linear growth and birth outcomes, especially in the presence of poor environmental conditions (Liberato, Singh, & Mulholland, 2013; Millward, 2017). Several observational and intervention studies have shown positive associations between ASF intake and linear growth even after adjusting for confounders such as socio‐economic status, morbidity profile, parental education, and nutritional status (Allen & Dror, 2011; Iannotti et al., 2017; Millward, 2017; Neumann, Harris, & Rogers, 2002). In further support, PDCAAS‐corrected quality protein intake showed positive impact on the rate of weight gain in severely acute malnourished Peruvian children of 6–32 months (Arsenault & Brown, 2017). A recent observational study of South Indian pregnant women showed a significant positive association (*P* < .001) between protein intake from milk products and birthweight (Mukhopadhyay et al., 2018). The state‐level analyses of rural NNMB dietary intake data show that, unlike plant source foods, the consumption of ASF in pregnant women has a significant inverse correlation with LBW prevalence (*r*s = −.72, *P* < .05). A similar inverse but non‐significant relation was observed between ASF intake and prevalence of stunting in children (*r*s = −.48, *P* = .13 for 1–3 years and *r*s = −.47, *P* = .14 for 4–6 years). It is worth noting that such inverse association can be attributed to different macronutrient and micronutrient contents in their diets and corroborates with the observed significant positive association between ASF intake and height for age in preschool children from developing countries of Central and South America in Demographic and Health Surveys (Ruel, 2012).

It is possible to improve the quality of protein intake with modest additions of ASFs to reduce the risk of quality protein (DIAAS corrected) inadequacy, but a DIAAS of 100% cannot be achieved unless safe environmental conditions are ensured for children. If not, an additional allowance of protein and IAA is required, which in turn raises the amino acid score of the requirement. Adding a combination of 100‐ml milk (1 glass) and 50‐g egg (1 egg) increased the DIAAS of the diet protein to 92% in 1–3 years and reduced the DIAAS‐corrected risk of inadequacy to 9%. Similarly, for 4–6 years, additional 200‐ml milk (2 glasses) and 50‐g egg increased the DIAAS of the diet to 92% and reduced the quality‐corrected risk of protein inadequacy to 5%. However, risk reduction is more difficult in pregnant women, where the addition of 350‐ml milk (3½ glasses) and 75‐g eggs (1½ pieces) increased the DIAAS of diet to 100% but reduced the risk of DIAAS‐corrected protein inadequacy only to 20%, because of a habitual low protein intake in this population. The addition of legumes was not as effective due to their poor digestibility; as with addition of 50 and 60 g of legume day^−1^, the risk of DIAAS‐corrected protein inadequacy reduced only to 20%, 19%, and 58% in children (1–3 and 4–6 years) and pregnant women, respectively. Plant source foods have other beneficial components like fibre and so should not be ignored as important foods, but from a quality protein‐provision perspective, they are inadequate. The addition of ASFs in above‐mentioned amounts increased the DIAAS‐corrected protein energy ratio by ~5% in children 1–3 (from 7.1% to 12.2%) and 4–6 years (from 6.6% to 11.5%) and (from 7.4% to 12.4%) pregnant women, which was within (<15%) the current recommendation (World Health Organization, Food and Agricultural Organization & United Nations University, 2007).

The present analyses have limitations related to the available data. First, the calculations of the IAA requirements during pregnancy are based on measurements from a single study performed in healthy pregnant Indian women. There are limited data available on metabolic adaptation during pregnancy. A mean pregnancy IAA score of the requirement was also constructed to compare with diets of Indian pregnant women at an unspecified time of pregnancy. Second, dietary data were based on a single day “total household” recall and not an age‐specific individual recall; to get to an individual consumption, age‐ and sex‐specific consumption units were applied (NNMB, 2012), which may lack representation of a regular, weekly, or monthly consumption of food groups. Third, the NNMB dietary intake data were surveyed for the 4‐ to 6‐year age group but correlated with the prevalence of stunting and underweight in children 3–5 years. Fourth, digestibility values for cereals and millets were taken from measurements made in children; these data were used for calculating the DIAAS of pregnancy protein intakes; similarly, egg digestibility values were assumed to be the same as that for milk and flesh foods.

The review presents for the first time to our knowledge the IAA requirements and scoring pattern for pregnant women, based on Indian data. It underscores the need for protein quality evaluation of commonly consumed foods and diets in population, specifically for vulnerable age groups and pregnancy, in a poor environmental setting. The state‐wise estimates of DIAAS‐corrected risk of protein inadequacy can be used for planning intervention trials, such that dietary interventions aiming to promote linear growth in young children and positive birth outcomes are optimised for the quality of their protein as well as safe environmental conditions.

## CONFLICTS OF INTEREST

The authors declare that they have no conflicts of interest

## CONTRIBUTIONS

SB and NS collated and analysed the data; NS and AVK conceived the research; and all authors contributed to the writing of the manuscript. NS had primary responsibility for the final content.
